# Claudin-18 expression is associated with poorer overall survival in colorectal cancer

**DOI:** 10.1371/journal.pone.0360740

**Published:** 2026-10-26

**Authors:** Aya Y. Al-Kabariti, Manal A. Abbas, Maxim Alashhab, Sneha Smarakan, Maher A. Sughayer

**Affiliations:** 1 Biopharmaceutics and Clinical Pharmacy, Faculty of Pharmacy, Al-Ahliyya Amman University, Amman, Jordan; 2 Department of Medical Laboratory Sciences, Faculty of Allied Medical Sciences, Al-Ahliyya Amman University, Amman, Jordan; 3 Department of Pathology and Laboratory Medicine, King Hussein Cancer Center, Amman, Jordan; 4 Institute of Cancer Therapeutics, Faculty of Health and Social Care, University of Bradford, West Yorkshire, United Kingdom; 5 Faculty of Medicine, The University of Jordan, Amman, Jordan; Aix-Marseille University, FRANCE

## Abstract

Claudin 18, a tight junction protein, has emerged as a therapeutic target in various gastrointestinal cancers, notably gastric adenocarcinoma. However, its prevalence and clinicopathological significance in colorectal cancer (CRC) remain incompletely understood. In the current single-center, retrospective, immunohistochemical study, the prevalence and prognostic value of Claudin-18 was investigated in CRC. Claudin-18 expression was detected in 15.9% (17/107) of CRC cases, with negative/weak expression in 84.1% (90/107). No significant associations were observed between Claudin-18 expression and age, sex, tumor site, T stage, lymph node status, distant metastasis, TNM stage, histological subtype, or lymphovascular invasion (all *p* > 0.05). No significant associations were identified between Claudin-18 expression and age, sex, tumor location, histological subtype, or TNM stage. Furthermore, Claudin-18 expression independently predicted survival outcomes in CRC where Kaplan–Meier analysis demonstrated significantly worse overall survival (OS) in patients with strong/moderate Claudin-18 expression compared with Claudin-18 -negative/weak expression (log rank test, *p* = 0.024). Mean OS was markedly longer in Claudin-18 -negative/weak expression group (121.0 vs.76.6 months). Progression-free survival (PFS) analysis demonstrated a trend toward poorer outcomes among patients with increased Claudin-18 expression; however, the difference did not reach statistical significance (log-rank test, *p* = 0.066). Event-free survival (EFS) was also significantly reduced in patients with strong/moderate Claudin-18 expression (log-rank test, *p* = 0.022). Mean EFS was markedly longer in CLDN18 negative/weak expression (93.1 vs. 31.1 months). Multivariable binary logistic regression showed that the overall model was statistically significant (χ2(7) = 22.657, p = 0.002). However, none of the evaluated variables—including gender, age, lymphovascular invasion, tumor laterality, TNM stage, MSI status, histological type, and Claudin-18 expression—was independently associated with the outcome (all p > .05). Claudin-18 expression was not a significant predictor in the adjusted model (p = 0.931; OR = 0.63). In conclusion, despite its low prevalence in the current cohort, Claudin-18 expression was associated with poorer overall survival; however, its independent prognostic value was not established.

## Introduction

Colorectal cancer (CRC) continues to be a leading cause of cancer morbidity and mortality worldwide, characterized by substantial molecular and clinical heterogeneity [1,2]. Despite advances in surgical techniques and the integration of molecularly targeted agents, the prognosis of advanced CRC remains poor, with 5-year survival below 20% for unresectable disease [3]. The ever-present need for novel biomarkers and therapeutic strategies underlines ongoing research into key molecular drivers of CRC progression [4].

Claudins are a family of 27 transmembrane proteins that serve as fundamental components of tight junctions in epithelial cells, regulating the permeability of ions and small molecules between cells [5]. Tight junction proteins, especially the claudin family, are critical to maintaining cellular polarity and barrier function in epithelial tissues. Dysregulation of various claudins has been implicated in carcinogenesis, tumor invasion, and metastasis. Two highly homologous isoforms, Claudin-18  .1 and Claudin-18, are produced by alternative splicing of the first exon of the Claudin-18 gene, which is found on chromosome 3q22 and has six exons [6]. Claudin-18 has four transmembrane domains, a cytoplasmic tail, and two extracellular loops. These isoforms exhibit tissue-specific expression: Claudin-18  .1 is primarily expressed in the lung, whereas Claudin-18 is primarily expressed in the stomach and functions as a highly selective marker of differentiated gastric epithelial cells. However, it is not present in the gastric mucosa’s stem cell zone [7]. Claudin 18 comprises two isoforms, Claudin-18 .1 and Claudin-18.2; the latter is primarily expressed in the gastric mucosa and becomes ectopically expressed in certain cancers, such as gastric, pancreatic, and pulmonary malignancies. The emergence of Claudin-18 as a therapeutic target is exemplified by clinical success in gastric cancers, where agents like zolbetuximab have demonstrated efficacy in Claudin-18 -positive tumors [8].

Notably, in a recent study, approximately 2% of CRC cases were CLDN18.2 positive and were associated with adverse clinicopathological features, including mucinous or poorly differentiated histology, advanced T3–4 stage, lymphatic invasion, BRAF V600E mutation, and deficient MMR status. However, CLDN18.2 positivity was not significantly associated with relapse-free or overall survival [9]. Given the ongoing development of Claudin-18 -targeted therapies and the global burden of CRC, there is a compelling rationale to further understand the biological and clinical implications of Claudin-18 expression in CRC to facilitate precision medicine approaches. Therefore, the current investigation studied the prevalence, clinicopathological associations, molecular features, and clinical implications of Claudin-18 expression in CRC. Our aim is to provide a focused update for clinicians and researchers, set in the context of evolving targeted therapies, and to highlight future directions necessary for optimal clinical integration.

## Materials and methods

### Ethical consideration

The present study was approved by the Institutional Ethics Committee of the KHCC, Amman, Jordan (Decision No. 24 KHCC 259). All the specimens used for the present study were archived paraffin tumor tissue specimens with no direct patient identifier. Medical history of the patient was extracted from the electronic medical records of the Department of Pathology at KHCC, Amman, Jordan.

Adult patients of Arabic ethnicity diagnosed with primary CRC during the period January 2011 to December 2021 were included in the study. All enrolled patients underwent surgical resection of the primary lesion at KHCC, Amman, Jordan. Patients with left-sided CRC received chemotherapy before surgery. The exclusion criteria included history of another neoplasm or not-well preserved or insufficient paraffin block.

### Tissue microarray construction

The tissue microarray (TMA) blocks were constructed from archival paraffin-embedded tissue specimens of primary CRC using a manual tissue microarrayer (Quik Ray) as previously described [10].

### Immunohistochemistry (IHC)

Four-μm-thick paraffin tissue sections were cut from TMA blocks. Antigen retrieval was performed using ULTRA CC1 cell conditioning solution (pH 8) for 64 minutes at 100 °C. Immunohistochemical staining for Claudin-18 was performed using an automated BenchMark ULTRA PLUS system (Roche, USA). Immunohistochemical staining for Claudin-18 was performed using the VENTANA Claudin-18 (43-14A) RxDx Assay (mouse monoclonal antibody, clone 43-14A; Ventana Medical Systems, Roche Diagnostics, Tucson, AZ, USA) for 16 minutes at 36 °C on a BenchMark ULTRA platform with the OptiView DAB IHC Detection Kit, according to the manufacturer’s instructions. Normal gastric mucosa was used as positive control.

Claudin-18 IHC expression in the primary tumors of CRC patients was evaluated using a light microscope (three 20× fields, Olympus, Tokyo, Japan) by an experienced pathologist (M.A.S.). Claudin-18 expression was evaluated as the percentage of tumor cells showing moderate (+2) to strong (+3) membranous staining, with a cutoff of ≥75% considered positive, in accordance with the manufacturer’s scoring algorithm. The strong (3+) intensity of staining is scored if intensity is approximately similar to the on-slide normal gastric tissue control (Fig 1G) while moderate staining (+2) is considered if staining is intermediate between weak or faint staining and strong staining in a manner similar to HER2 scoring in terms of intensity. The weak to moderate staining is exemplified in Figs 1A and 1B.

**Fig 1:**
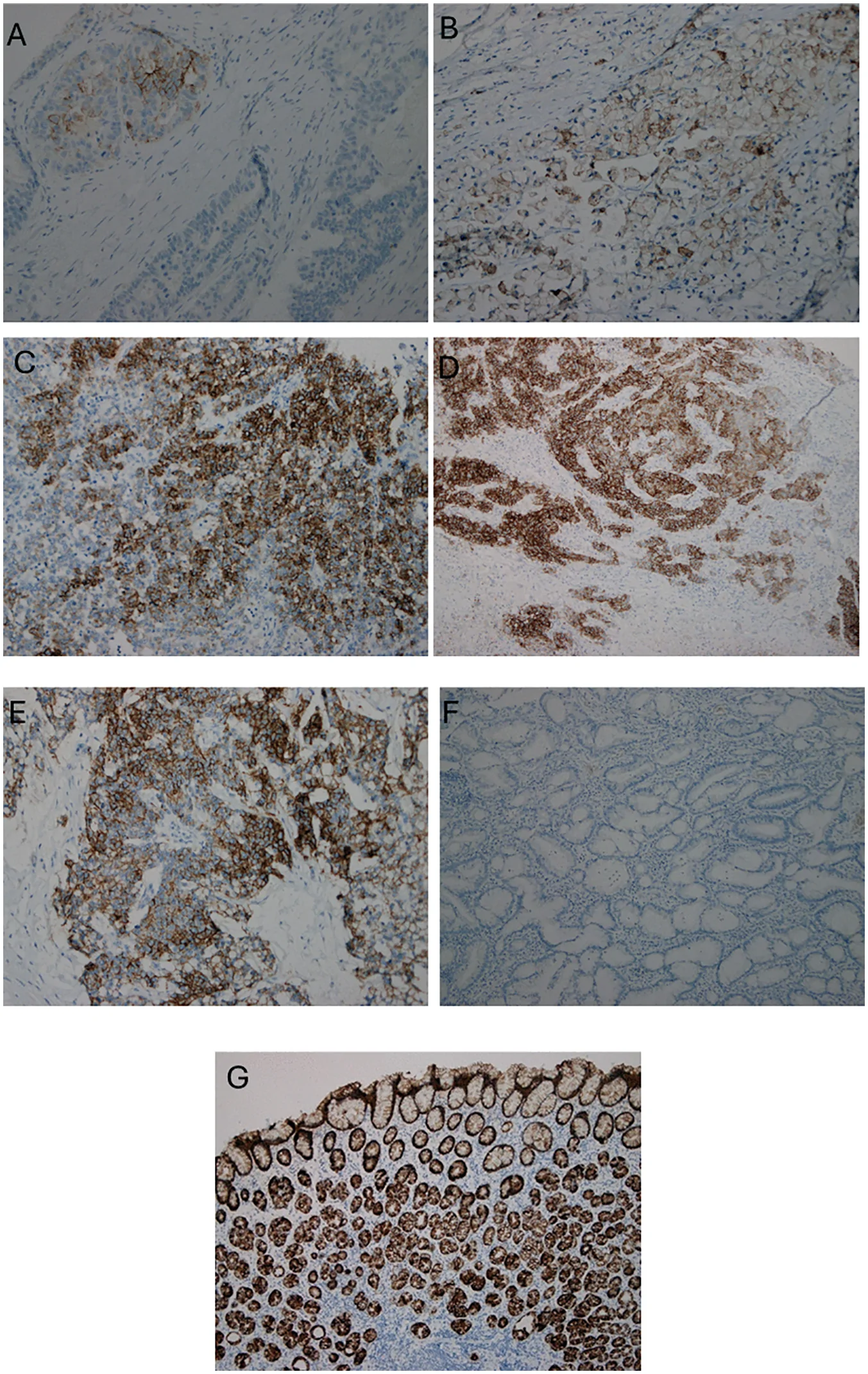
Patterns of Claudin-18 expression in CRC. Claudin-18 expression was scored based on the percentage and intensity of membranous staining in tumor cells on 20x fields as follows: (A) 20% weak to moderate Claudin-18 expression. (B) 80% weak to moderate Claudin-18 expression in signet ring cancer. (C) 90% moderate to strong Claudin-18 expression. (D) 95% strong to moderate Claudin-18 expression. (E) 95% strong to moderate Claudin-18 expression. (F) Negative control. (G) normal gastric mucosa showing strong (3+) membranous staining in100% of the cells serving as a positive control. A positive reaction appears as a brown color at the sites of Claudin-18 expression, while a negative reaction appears blue in color (hematoxylin stained).

### Tumor classification

Colorectal tumors were classified as right-sided or left-sided based on their anatomical location. Right-sided tumors, included those arising in the cecum, ascending colon, hepatic flexure, and transverse colon. In contrast, left-sided tumors are located in the splenic flexure, descending colon, sigmoid colon, rectosigmoid junction, and rectum. For TNM, AJCC (8th edition) stage grouping was used [11].

### Statistical analysis

Demographic and clinicopathological data of CRC patients were presented as counts (n) and percentages (%). Fisher’s exact tests were used to find the relationship between Claudin-18 IHC expression and demographic and clinicopathological features of the study specimens. The Kaplan-Meier models were utilized to calculate overall survival rate for CRC patients and the log-rank chi-square test was utilized for comparisons. Overall survival (OS) of CRC patients was assessed from the time of diagnosis of the primary lesions from CRC to the time of death or the last visit. Progression-free survival (PFS) was calculated from the time of diagnosis to the time of relapse or the last visit while event-free survival (EFS) was calculated from the time of diagnosis to the first documented event, including disease progression, recurrence, or death from any cause, or to the last follow-up for censored patients. Multivariable binary logistic regression analysis was performed to identify factors independently associated with overall survival. All variables included in the model were entered simultaneously. Hazard ratios (HRs), 95% confidence intervals (CIs), and corresponding p values were calculated for each variable. All candidate predictors were entered simultaneously into the regression model using the Enter method. The independent variables included gender, age (years), lymphovascular invasion, tumor laterality (right versus left), TNM stage, microsatellite instability (MSI) status, histological type, and Claudin-18 expression. Categorical variables were coded using appropriate reference categories, while age was analyzed as a continuous variable. All statistical analyses were performed using Statistical Package for the Social Sciences (SPSS) version 26 *software* (SPSS Inc., Chicago, *Illinois, United States).* Two-sided *p* values ≤0.05 were considered statistically significant.

## Results

### Clinicopathological characteristics of the patients

A total of 107 primary CRCs were studied, with a median age of 56 years (range, 18–86 years). Clinicopathological characteristics of the study cohort are summarized in Table 1 and the supplementary data. Among the 107 CRC cases, 59 (55.1%) were >56 years and 48 (44.9%) were ≤56 years, with no significant difference in age between the negative/weak and strong/moderate Claudin-18 groups (*p* = 0.596). Males comprised 72 (67.3%) cases and females 35 (32.7%), with comparable distributions between the two expression groups (*p* = 0.785). Tumor site was available for 102 cases, including 52 (51.0%) right-sided and 50 (49.0%) left-sided tumors (*p* = 0.150). T3 was the most frequent T stage (70, 65.4%), followed by T4 (32, 29.9%), with no significant difference between expression groups (*p* = 0.112). Regarding lymph node status, 48 (44.9%) were N0, 29 (27.1%) N1, 27 (25.2%) N2, and 3 (2.8%) N3 (*p* = 0.406). M0 disease was present in 96 (89.7%) cases and M1 in 11 (10.3%) (*p* = 0.376). TNM stages II and III were most frequent, accounting for 42 (39.3%) and 49 (45.8%) cases, respectively (*p* = 0.545). Among 105 cases with available histological data, moderately differentiated adenocarcinoma was the most common subtype (81, 77.1%), followed by poorly differentiated (19, 18.1%) and mucinous adenocarcinoma (5, 4.8%) (*p* = 0.084). Lymphovascular invasion was absent in 60 (56.1%) and present in 48 (44.9%) cases, with no significant difference between the Claudin-18 expression groups (*p* = 0.109).

**Table 1:** Demographic and clinicopathologic features of Claudin-18 positive and negative cases with CRC.

Characteristic	Total n (%)	Negative/weak Claudin-18 expression (n = 90)	Strong/moderate Claudin-18 expression (n = 17)	*p*-value of Chi-square test
Age (years) (n = 107)				0.596
>56 (median)	59 (55.1%)	51 (56.7%)	8 (47.1%)	
≤56	48 (44.9%)	39 (43.3%)	9 (52.9%)	
Gender (n = 107)				0.785
Female	35 (32.7%)	29 (32.2%)	6 (35.3%)	
Male	72 (67.3%)	61 (67.8%)	11 (64.7%)	
Tumor site (n = 102)				0.150
Right-sided	52 (51.0%)	42 (47.7%)	10 (71.4%)	
Left-sided	50 (49.0%)	46 (52.3%)	4 (28.6%)	
Tumor T stage (n = 107)				0.112
T1	1 (0.9%)	0 (0.0%)	1 (5.9%)	
T2	4 (3.7%)	4 (4.4%)	0 (0.0%)	
T3	70 (65.4%)	61 (67.8%)	9 (52.9%)	
T4	32 (29.9%)	25 (27.8%)	7 (41.2%)	
Lymph node status (n = 107)				0.406
N0	48 (44.9%)	41 (45.6%)	7 (41.2%)	
N1	29 (27.1%)	26 (28.9%)	3 (17.6%)	
N2	27 (25.2%)	20 (22.2%)	7 (41.2%)	
N3	3 (2.8%)	3 (3.3%)	0 (0.0%)	
Metastasis (n = 107)				0.376
M0	96 (89.7%)	82 (91.1%)	14 (82.4%)	
M1	11 (10.3%)	8 (8.9%)	3 (17.6%)	
TNM stage (n = 107)				0.545
I	5 (4.7%)	4 (4.4%)	1 (5.9%)	
II	42 (39.3%)	37 (41.1%)	5 (29.4%)	
III	49 (45.8%)	41 (45.6%)	8 (47.1%)	
IV	11 (10.3%)	8 (8.9%)	3 (17.6%)	
Histological subtype (n = 105)				0.084
Moderately differentiated adenocarcinoma	81 (77.1%)	70 (80.5%)	10 (58.8%)	
Poorly differentiated adenocarcinoma	19 (18.1%)	14 (16.1%)	5 (29.4%)	
Mucinous adenocarcinoma	5 (4.8%)	3 (3.4%)	2 (11.8%)	
Lymphovascular invasion (n = 107)				0.109
Absent	60 (56.1%)	54 (60.0%)	6 (35.3%)	
Present	48 (44.9%)	37 (41.1%)	11 (64.7%)	

Negative/weak Claudin-18 expression as detected in 90 out of 107 cases while 17 cases showed strong/moderate expression (15.9%). Histologically, Claudin-18 showed membranous localization (Fig 1). Strong/moderate Claudin-18 expression showed no significant association with age, sex, tumor site, TNM stage, lymphovascular invasion or histological subtype (Table 1).

In the multivariable logistic-regression model, none of the included variables was independently associated with the outcome, as all Wald-test p values exceeded 0.05. Claudin-18 expression was not associated with the outcome (B = −0.463, SE = 5.384, Wald = 0.007, CI: 1.64 × 10 − 5–24,188.99, p = 0.931; OR = 0.630).

Molecular analysis was available for a subset of patients. Kirsten rat sarcoma viral oncogene homolog (KRAS) mutational status was assessed in 26 cases, of which 8 patients (30.8%) harbored the G12D (Gly12Asp) mutation and 2 patients (7.7%) had the A146V variant, while 16 patients (61.5%) were KRAS wild type. Microsatellite instability (MSI) testing was performed in 21 cases, revealing alterations in MLH1 in 7 patients (38.1%), MSH2 in 3 patients (14.3%), MSH6 in 3 patients (14.3%), and PMS2 in 8 patients (33.3%). B-Raf proto-oncogene, serine/threonine kinase (BRAF) mutation analysis, available for 9 patients, identified BRAF mutations in 2 cases (22.2%), whereas 7 patients (77.8%) were wild type. Neuroblastoma; RAS: viral oncogene homolog (NRAS) status was evaluated in 4 patients, all of whom were wild type with no NRAS mutations detected. No significant associations were found between the presence of any of these mutations and the positivity of Claudin-18.

### Survival analysis

Patients with strong/moderate Claudin-18 expression demonstrated significantly shorter overall survival (OS) compared with those with negative or weak expression. The mean OS time was 76.6 months (95% CI: 34.4–118.7) in the strong/moderate expression group, compared with 121.0 months (95% CI: 100.6–141.4) in the negative or weak expression group. Median OS was 25.0 months (95% CI: 0.0–92.1) for patients with strong/moderate Claudin-18 expression, whereas the median survival for the negative or weak expression group was not reached. Kaplan–Meier analysis demonstrated a significant difference in survival distributions between the two groups (log-rank test, *p* = 0.024), indicating that increased Claudin-18 expression was associated with poorer OS outcomes in CRC (Fig 2A-B).

**Fig 2:**
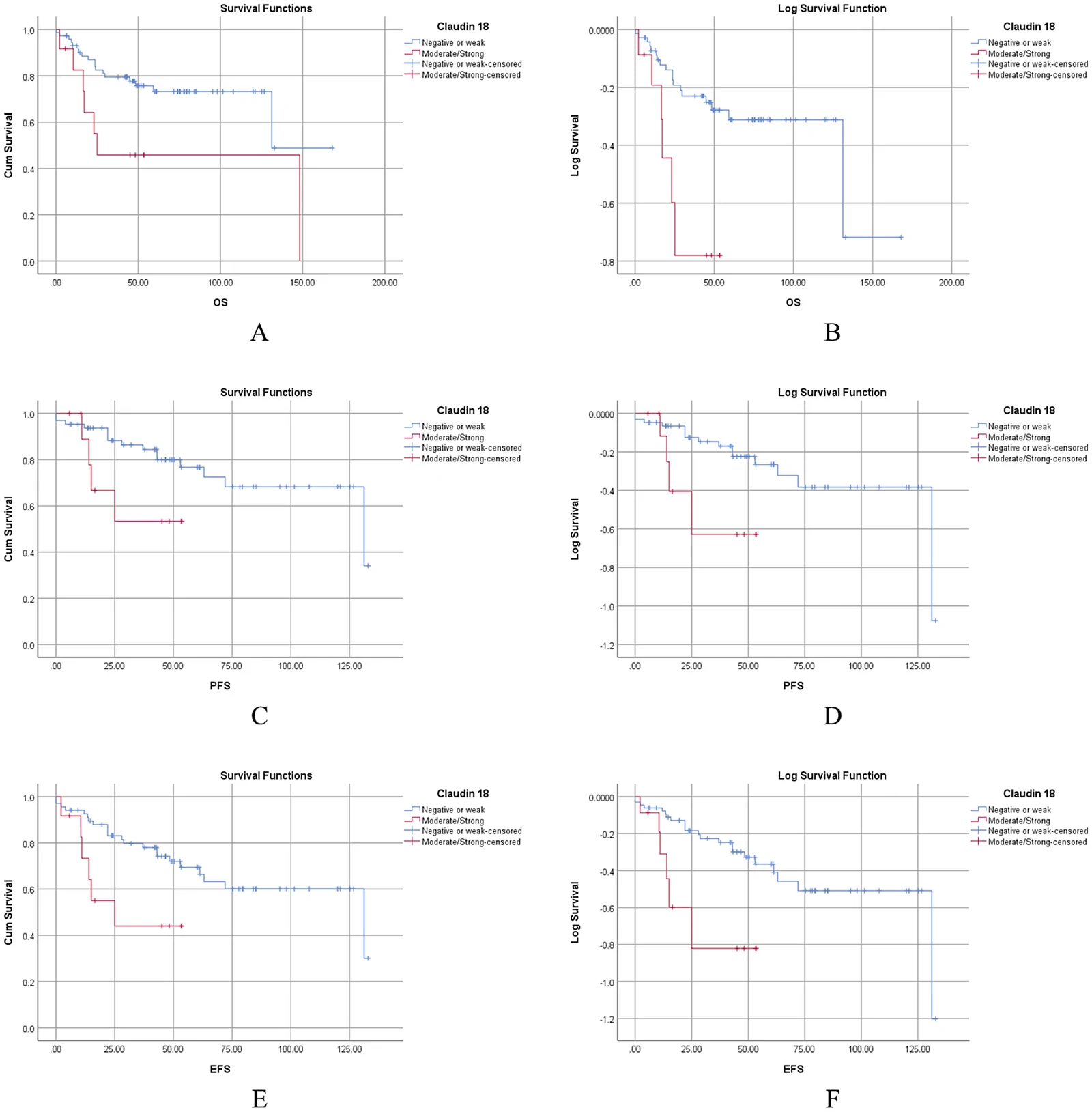
Kaplan-Meier test. (A) Overall survival. (B) Log overall survival. (C) Progression-free survival. (D) Log Progression-free survival. (E) Event-free survival. (F) Log event-free survival.

Patients with strong/moderate Claudin-18 expression showed shorter progression-free survival (PFS) compared with those with negative or weak expression. The mean PFS time was 36.3 months (95% CI: 23.8–48.9) in the strong/moderate expression group, compared with 102.1 months (95% CI: 88.5–115.7) in the negative or weak expression group. The median PFS time for patients with negative or weak Claudin-18 expression was 131.2 months (95% CI: 48.1–214.2), whereas the median survival for the strong/moderate expression group could not be estimated. Kaplan–Meier analysis demonstrated a trend toward poorer PFS among patients with increased Claudin-18 expression; however, the difference did not reach statistical significance (log-rank test, *p* = 0.066) (Fig 2C-D).

Event-free survival (EFS) survival analysis demonstrated significantly poorer outcomes among patients with strong/moderate Claudin-18 expression compared with those showing negative or weak expression. The mean EFS time was 31.1 months (95% CI: 18.8–43.4) in the strong/moderate expression group, compared with 93.1 months (95% CI: 79.6–106.6) in the negative or weak expression group. Median EFS was 25.0 months (95% CI: 0.0–52.6) for patients with strong/moderate Claudin-18 expression, whereas the median EFS for the negative or weak expression group was 131.2 months (95% CI: 48.0–214.4). Kaplan–Meier analysis revealed a statistically significant difference in survival distributions between the two groups (log-rank test, *p* = 0.022), indicating that increased Claudin-18 expression was associated with poorer EFS survival in CRC patients (Fig 2E-G).

## Discussion

It is well-established that normal colonic tissue does not express Claudin-18 [12]. In CRC, Claudin-18 was expressed in 15.9% (17/107) of cases in the present study. Other studies reported lower Claudin-18 expression of 2.1% [9], 3.4% (40/1175) [4], 0.9% (2/203) [13] in CRC adenocarcinoma. On the other hand, a higher prevalence of 35% was reported in sessile serrated adenoma/polyps [14] and 27.8% of serrated adenocarcinomas [15]. In another study, no membranous Claudin-18 staining was detected in hyperplastic polyps or adenomas, whereas it was observed in 44.4% of sessile serrated adenomas and 12.8% of traditional serrated adenomas [16].

The findings of the present study may be interpreted in the context of dynamic epithelial tight-junction remodeling during CRC carcinogenesis. Claudin proteins are not static structural components but undergo context-dependent changes in expression and localization during malignant transformation, influencing epithelial polarity, cell–cell adhesion, and paracellular permeability [15]. Disruption of this tightly regulated network may promote epithelial plasticity and facilitate epithelial–mesenchymal transition (EMT), characterized by loss of epithelial adhesion and polarity and acquisition of increased motility and invasiveness [15]. In inflammation-associated CRC, persistent mucosal inflammation may further disrupt tight-junction integrity and alter claudin expression, increasing epithelial permeability and exposure to luminal microbial products. This can amplify inflammatory signaling, epithelial injury, and regenerative responses, potentially contributing to dysplasia and malignant transformation within chronically inflamed mucosa [17]. Within this dynamic claudin landscape, CLDN18.2 may represent a distinct epithelial phenotype rather than simply a marker of tumor progression. Recent CRC studies have shown that, although CLDN18.2 expression is uncommon, it is enriched in tumors with specific histological and molecular characteristics, including proximal location, mucinous differentiation, mismatch-repair deficiency, and BRAF V600E mutation [9]. Therefore, its expression may identify a biologically distinct subset of CRC.

Previous studies reported that mucinous adenocarcinoma expresses higher Claudin-18 as reported by one study (27%) compared to 2% in CRC adenocarcinoma [18]. Colorectal mucinous adenocarcinoma comprised only 4.8% of the present cohort while signet-ring, and serrated subtypes represented only 3/107 cases (2.8%). Therefore, larger studies are needed to confirm this finding. It was suggested that Claudin-18 expression in CRC may mark a “gastric-like” molecular phenotype, supported by observed predisposition to mucinous differentiation and co-expression with markers such as MUC5AC [9].

In the present study, Claudin-18–positive CRCs showed a higher frequency of lymphovascular invasion, although this association did not reach statistical significance (*p* = 0.109). On the other hand, no significant associations were identified between Claudin-18 expression and age, sex, tumor location, histological subtype, or TNM stage. In a recent study involving 343 rectal cancer patients who underwent concurrent chemoradiotherapy, elevated Claudin-18 expression was associated with older age, higher pre-concurrent chemoradiotherapy tumor N stage, higher post-concurrent chemoradiotherapy tumor T stage and more vascular invasion [19]. Also, Claudin-18 expression in CRC correlated with poorly differentiated histology, high frequency of advanced T stage, right-sided tumor location and pronounced lymphatic invasion [9]. In serrated adenocarcinoma, Claudin-18 expression correlated with more extensive lymph node metastasis and advanced overall stage [16]. The lack of standardization of immunohistochemical staining procedure and scoring systems may account to differences between studies.

In the present investigation, Claudin-18 expression was not independently associated with the outcome (adjusted OR = 0.63, p = 0.931), despite a significant overall model fit (p = 0.002). Additionally, Claudin-18 staining was found to be associated with worse overall survival and event-free survival in CRC. This agrees with the results of a previous study in which Claudin-18 expression was found to be an independent predictor of survival in CRC [20]. In another study, upregulated Claudin-18 expression was associated with worse disease-free survival, local recurrence-free survival and metastasis-free survival in rectal cancer taking concurrent chemoradiotherapy [19].

Several methodological considerations should also be acknowledged. Assessment of a single tissue-microarray core may underestimate intratumoral heterogeneity and focal CLDN18.2 expression. Moreover, the ≥ 75% cutoff used in our study was established primarily for therapeutic selection in gastric/gastroesophageal adenocarcinoma and has not been validated as a prognostic threshold in CRC. Finally, only 17 of 107 cases (15.9%) were CLDN18.2-positive, limiting statistical power and the precision of survival estimates. Thus, our findings should be considered hypothesis-generating and require validation in larger, multicenter CRC cohorts using standardized CLDN18.2 assessment.

## Conclusion

Claudin-18 expression was detected in a subset of colorectal cancers in this single-center retrospective cohort, with a prevalence of 15.9%. Although relatively infrequent, Claudin-18 emerged as an independent prognostic marker, as patients with strong to positive Claudin-18 -expressing tumors exhibited significantly worse overall survival compared with Claudin-18 -negative or weak expression. Large, multicenter studies are warranted to validate these results and to clarify the role of Claudin-18 -targeted therapies in CRC.

## Supporting information

S1 Data Raw data of research cases showing results of Claudin 18 staining along with survival and event data. (XLSX)
